# Pharmacokinetics and metabolism of icaritin in rats by UPLC‐MS/MS

**DOI:** 10.1002/fsn3.1263

**Published:** 2019-11-12

**Authors:** Zhen‐Wu Huang, Yue‐Xin Yang, Ling‐He Huang, Shuang‐Qing Zhang

**Affiliations:** ^1^ National Institute for Nutrition and Health Chinese Center for Disease Control and Prevention Beijing China; ^2^ Woods Worth College University of Toronto Toronto ON Canada

**Keywords:** glucuronidated icaritin, icaritin, metabolism, pharmacokinetics, UPLC‐MS/MS

## Abstract

Icaritin (ICT) has distinct bioactivities, especially known for its beneficial effects on bone‐related degenerative disorders; however, its pharmacokinetic properties remain unknown. A novel developed UPLC‐MS/MS method for the determination of ICT and its main metabolite glucuronidated icaritin (GICT) was firstly applied to pharmacokinetic and metabolism studies of ICT in female rats, which were intraperitoneally given 40 mg/kg ICT. Following the protein precipitation of plasma samples with acetonitrile, ICT and GICT were separated on a C18 column using gradient elution mode and quantified in the multiple reaction monitoring mode. The linearities were acceptable for ICT (*r* = 0.9960) and GICT (*r* = 0.9968), and the lower limit of quantification values was 0.5 and 5 ng/ml, respectively. The accuracy fell in the range of 92.0%–103.1% and precisions were within 9.5%. Good linearity, accuracy, precision, and recovery were achieved for the UPLC‐MS/MS method. ICT was predominantly and rapidly biotransformed to GICT which was slowly eliminated in vivo with a terminal half‐life value of 4.51 hr. Pharmacokinetics of pure ICT eliminated biotransformation interference of *Epimedium* extract and disclosed genuine pharmacokinetic manner of ICT, as well as firstly elucidated low concentration and bioavailability of ICT in rat plasma.

## Graphical abstract

A major metabolite of the intraperitoneally administered icaritin in rat by intraperitoneal was firstly identified and quantified UPLC‐MS/MS for assay of icaritin, and its metabolite in plasma was established first paper reporting pharmacokinetics of icaritin and its metabolite

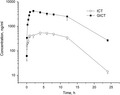

## 1 INTRODUCTION

**Figure 1:**
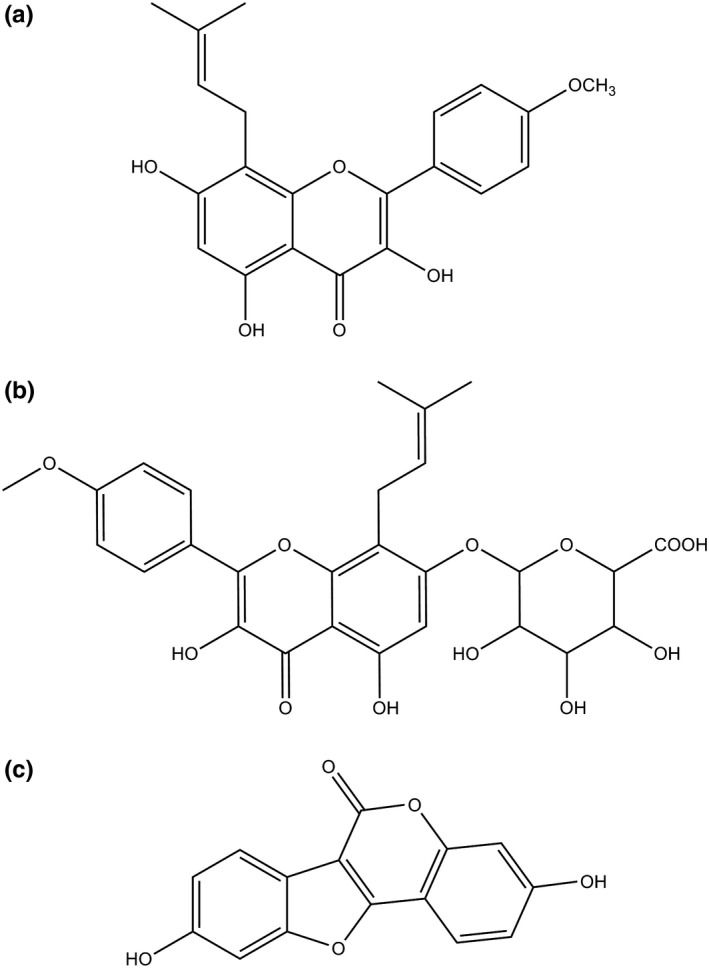
Chemical structures of ICT (a), GICT (b), and coumestrol (c)

Kidney‐tonifying herb *Epimedium*, as a dominate ingredient of several classic Chinese formulas, has been used to treat osteoporosis and other bone‐related degenerative disorders in China for over 20 centuries (Li, C., Li, Mei, & Lu, 2015). Among identified compounds from *Epimedium*, flavonoids are the principal bioactive components including icaritin, icariin, icariside, epimedin, and sagittatoside (Ma et al., 2011). Icaritin (ICT, Figure 1a) is a major flavonoid component in *Epimedium* and exerts many pharmacological activities, including anticancer (Huang, X., Zhu, & Lou, 2007; Li, S. et al., 2013; Sun, Chen, Qu, Wu, & Si, 2013; Tiong et al., 2012; Tong et al., 2011), anti‐inflammation (Lai et al., 2013), osteoprotective effect (Huang, J., Yuan, Wang, Zhang, & Wang, 2007), and reproductive function (Chen, M., Hao, Yang, & Li, 2014)). Moreover, the excellent osteogenic effects of ICT are assumed to prevent osteoporosis (Chen, S. et al., 2018; Huang, L. et al., 2018).

Although there have been several publications regarding the measurement of plasma ICT concentration after the intragastric administration of a crude *Epimedium* extract (Shen, Wong, Li, & Yong, 2009; Shen, Wong, & Yong, 2007; Wong, Shen, Lee, Li, & Yong, 2009), pharmacokinetic properties of pure ICT were quite different from the crude *Epimedium* extract, in which, besides ICT, also contained epimedin, icariside, and icariin. More than those, icariin was observed to be transformed to ICT, icariside I, and icariside II in rats (Shen et al., 2009), which resulted in increasing plasma ICT concentration after the administration of *Epimedium brevicornu* extract to rats (Shen et al., 2009; Wong et al., 2009); the other component, epimedin, was also metabolized to ICT in vivo that elevated plasma ICT level (Han, Shan, Zhou, Zhang, & Hou, 2013). Only one investigation reported the pharmacokinetics of pure ICT in rats using an ultraviolet detection. Unfortunately, glucuronidase and sulphatase were added to plasma to result in the conversion of phase II metabolites to ICT, and faulty pharmacokinetic parameters and bioavailability were obtained by total ICT (free ICT and released ICT) (Chang et al., 2012). Phase II enzymes were also employed to determine ICT concentration after *Epimedium brevicornu* extract was treated to rats (Wong et al., 2009). In our preliminary study, a phase II metabolite glucuronidated icaritin (GICT, Figure 1b) was found to be the major metabolite of ICT following the administration of pure ICT to rats.

Based on the results by Wong et al. (2009) and Chang et al. (2012), it was inferred that the exposure of phase II metabolites was several hundred times than that of ICT itself, and it was imperative to study the major metabolite of ICT for the safety assessment according to the guideline “safety testing of drug metabolites: guidance for industry (FDA, 2016).” In the present study, a reliable method based on UPLC‐MS/MS was firstly established for the simultaneous measurement of plasma ICT and GICT using protein precipitation and was used to study pharmacokinetics and metabolism of pure ICT in rats for exploring the fate of ICT in vivo and further supporting safety evaluation of ICT.

## 2 MATERIALS AND METHODS

### 2.1 Materials

ICT was obtained from Melonepharma Biotech Co. Ltd (Dalian, China). GICT was synthesized. Formic acid, ammonium formate, internal standard (IS) coumestrol (Figure 1c), and Cremophor EL were provided by Sigma (St. Louis, MO, USA). HPLC‐grade acetonitrile (ACN) and water were purchased from Merck (Darmstadt, Germany).

### 2.2 Instrumentation and experimental conditions

A Waters Xevo TQS UPLC‐MS/MS system was employed for the sample analysis, and MassLynx version 4.1 software was used for the data analysis. Elution A (water with 2 mmol/L ammonium formate and 0.05% formic acid, pH 3.0) and elution B (95% ACN/5% water containing 2 mmol/L ammonium formate and 0.05% formic acid) were pumped at 0.3 ml/min using a linear gradient program, as follows: 0–1.5 min, 90% A; 1.5–2.2 min, 90 to 10% A; 2.2–5.0 min, 10% A; 5.0–5.5 min 10 to 90% A; 5.5–6.5 min 90% A. The multiple reaction monitoring (MRM) *m*/*z* values of 367.1 → 297.1, 543.3 → 367.1, and 267.0 → 211.1 were used for the quantification of ICT, GICT, and coumestrol, respectively. The optimized conditions in the negative electrospray ionization (ESI) mode were cone of 60 V, capillary of 3.5 kV, desolvation temperature of 300°C, source temperature of 150°C, desolvation gas (N_2_) of 600 L/h, cone gas (N_2_) of 50 L/h, and collision gas (Ar) of 0.11 ml/min. Collision energy values of ICT, GICT, and coumestrol were 29, 20, and 27 eV, respectively.

### 2.3 Preparation of calibration standards and quality control samples

According to our previous method (Zhang, 2016), the concentrations of calibration standards (GICT/ICT) were 2/0.2, 5/0.5, 10/1, 20/2, 50/5, 80/8, 100/10, 150/15, and 200/20 ng/ml, and quality control (QC) samples, the lower limit of quantification (LLOQ), low QC (LQC), middle QC (MQC), high QC (HQC), and the upper limit of quantification (ULOQ) were also prepared at the levels of 2/0.2, 5/0.5, 80/8, 150/15, and 200/20 ng/ml, respectively.

### 2.4 Sample preparation

Each plasma sample taken from rats was successively spiked with 10 μl of IS working solution (1 μg/ml) and 200 µl of ACN, and subsequently was stirred by a vortex for 5 min and centrifuged at 10,000 g for 10 min at room temperature. The supernatant was collected with a 200‐µl polypropylene sample vial for the UPLC‐MS/MS analysis. Plasma samples with high ICT/GICT above the ULOQ were diluted by control plasma collected from rats without ICT injection to the concentration range of calibration curve and were measured repeatedly.

### 2.5 Method validation

According to the “ Bioanalytical Method Validation—Guidance for Industry” of the USA Food and Drug Administration (USFDA, 2018), the bioanalytical method of ICT and GICT in plasma was fully validated.

### 2.6 Pharmacokinetics

Six adult female Sprague Dawley rats, weighing 302 ± 21 g, were already catheterized in the right jugular vein and were intraperitoneally given at a dose of 40 mg/kg of ICT, which was prepared in a combined solvent containing Cremophor EL: ethanol: PEG 400: saline (13:7: 40:40, *v*/*v*). At 0.0333, 0.0833, 0.25, 0.5, 0.75, 1, 2, 4, 6, 8, 12, and 24 hr, approximately 0.25 ml of blood was taken from rats and was centrifuged to separate plasma and stored at −80°C within a week.

## 3 RESULTS AND DISCUSSION

### 3.1 Selectivity

**Figure 2:**
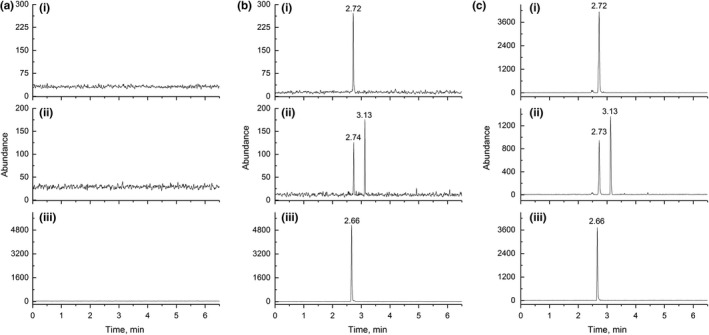
Representative MRM chromatograms (I: 543.3 → 367.1 for GICT at retention time of 2.72 min; II: *m*/*z* 367.1 → 297.1 for ICT at retention time of 3.13 min; III: *m*/*z* 267.0 → 211.1 for coumestrol at retention time of 2.66 min) for a blank plasma (a); a plasma spiked with ICT and GICT at LLOQ level (b); a plasma sample obtained 2 hr after intraperitoneal administration of ICT at a single dose of 40 mg/kg (c)

Representative chromatograms of control plasma and spiked plasma at LLOQ concentration of ICT and GICT are illustrated in Figure 2. The retention times of ICT, GICT, and the IS were 3.13, 2.72, and 2.66 min, respectively, and interferences for three analytes were not observed, indicating that this method was selective.

### 3.2 Linearity and LLOQ

Calibration curves of the ratios of peak areas of ICT/GICT to coumestrol against the corresponding concentration were established by a least‐squares linear regression. The representative calibration curves for ICT and GICT in the range of 0.2–20 and 2–200 ng/ml were y = 0.039881x−0.001495 and y = 0.010821x−0.014238, respectively. The values of correlation coefficient for ICT and GICT were 0.9960 and 0.9968, respectively. The deviation of all standards ranged among 3.4%–8.9%, and LLOQ values of ICT and GICT were 0.2 and 2 ng/ml, respectively.

### 3.3 Accuracy and precision

The accuracy of both ICT and GICT ranged from 92.0% to 103.1%, and precisions were within 9.5%, as shown in Table 1, indicating that the UPLC‐MS/MS method was reproducible.

**Table 1:** Accuracy, precision, matrix effect, and recovery of the method (Mean ± *SD*,* n* = 6)

Analyte	Concentration (ng/ml)	Intraday (%)		Interday (%)		Matrix effect (%)	Recovery (%)
		Accuracy	RSD	Accuracy	RSD		
ICT	0.2	103.1	5.3	100.4	6.7	/	/
	0.5	97.1	6.4	96.0	9.5	99.3 ± 10.1	93.1 ± 9.5
	8	100.1	7.0	102.3	7.4	97.5 ± 9.6	94.6 ± 8.4
	15	96.5	2.4	98.5	2.6	98.1 ± 5.3	96.2 ± 8.7
GICT	2	98.3	9.1	103.0	6.6	/	/
	5	95.2	2.0	97.8	6.6	97.4 ± 11.2	94.8 ± 8.9
	80	94.8	5.3	92.0	4.5	99.3 ± 9.1	97.6 ± 6.5
	150	102.6	5.7	92.9	6.8	98.2 ± 7.4	95.8 ± 6.6

Note

/, not performed.

### 3.4 Recovery and matrix effect

As illustrated in Table 1, the recoveries for ICT at the levels of LQC, MQC, and HQC were 93.1%, 94.6%, and 96.2%, while those for GICT were 94.8%, 97.6%, and 95.8%, respectively, with the RSD of less than 9.5% for ICT and 8.9% for GICT. In the case of matrix effect, RSD values at three levels were 5.3%–10.1% for ICT and 7.4%–11.2% for GICT. The recovery and matrix effect values of the IS were 81.3 ± 3.6% and 92.2 ± 3.3%, respectively.

### 3.5 Carryover and dilution integrity

Carryover was not found in blank plasma samples injected immediately following the analysis of the ULOQ samples in three independent runs. To verify the dilution integrity, samples with GICT/ICT concentrations of 10/1, 5/0.5, 2/0.2, and 1/0.1 μg/ml were diluted 100, 50, 20, and 10 times with blank samples and their determined levels were analyzed with the freshly prepared calibration curve. The accuracy values were 95.2%–102.6% for GICT and 96.2%–103.8% for ICT, while the precision levels were within 8.9% for GICT and within 11.6% for ICT.

### 3.6 Stability

The stability results are presented in Table 2. When plasma ICT/GICT was subject to three freeze‐thaw cycles, to postextraction storage for 6 hr at ambient temperature, to 4 hr in unprocessed sample at ambient temperature, and storage at −80°C for 7 days, the accuracy of ICT fell in the range of 94.2%–98.3% and that of GICT shifted within 89.9%–104.3%, as well as the precisions were within 10.2% and 6.9% for ICT and GICT, respectively, indicating that ICT and GICT were stable during sample preparation and 7‐day storage at −80°C.

**Table 2:** Stability results for ICT and GICT in rat plasma. Data are expressed in % (*n* = 5)

Stability conditions	ICT				GICT			
	0.5 ng/ml		15 ng/ml		5 ng/ml		150 ng/ml	
	Accuracy	RSD	Accuracy	RSD	Accuracy	RSD	Accuracy	RSD
3 freeze‐thaw cycles	94.2	6.7	99.1	5.1	89.9	5.4	91.2	5.3
Postpreparative	97.4	10.2	94.3	6.9	98.1	10.2	104.3	4.7
Short term	97.1	6.9	98.7	5.5	95.4	7.7	96.4	6.1
Long term	98.3	8.1	98.9	5.7	96.5	7.5	95.8	6.9

### 3.7 Pharmacokinetics

**Figure 3:**
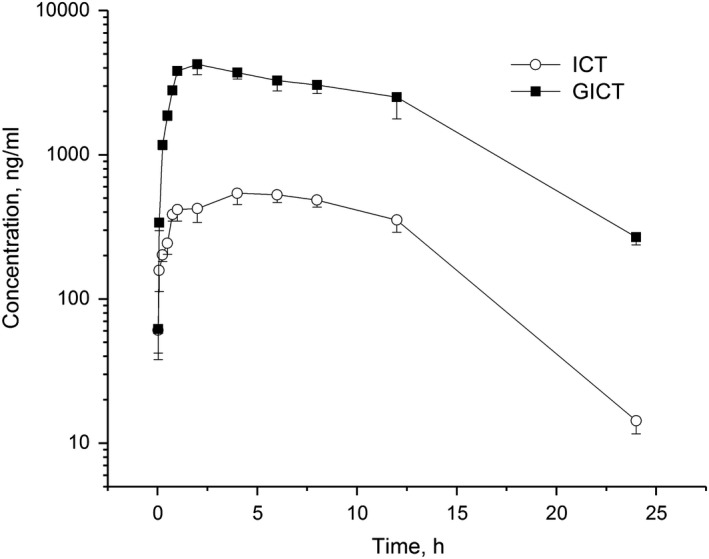
The mean plasma concentration–time profiles of ICT and GICT after intraperitoneal administration of ICT to rats at a single dose of 40 mg/kg (*n* = 6)

Figure 2c depicts the chromatogram of a plasma sample obtained 2 hr after intraperitoneal administration of ICT, and Figure 3 illustrates the pharmacokinetic profiles of ICT and GICT in rat plasma. The pharmacokinetic parameters of ICT itself and GICT were estimated by the noncompartment model of WinNonlin software 5.2.1, as shown in Table 3.

**Table 3:** The pharmacokinetic parameters of ICT and GICT after a single intraperitoneal administration of ICT to rats at a dose of 40 mg/kg. Values are presented as mean ± *SD* (*n* = 6)

Parameter	Unit	ICT	GICT
t_1/2, λz_	hr	3.14 ± 0.34	4.51 ± 0.65
AUC_0−24 hr_	hr ng/ml	7,937 ± 442	47,011 ± 6,641
V	L/kg	22.79 ± 3.01	5.69 ± 1.53
Cls	L/hr/kg	5.43 ± 0.85	1.02 ± 0.17
MRT_0−24 hr_	hr	7.55 ± 0.97	7.56 ± 0.70

Note

t_1/2, λz_, terminal half‐life; AUC_0–24 hr_, area under curve from 0 to 24 hr; Cls, systemic clearance; V, volume of distribution; MRT_0–24 hr_, mean residence.

Poor aqueous solubility and high lipophilicity of ICT resulted in its poor bioavailability (Zhang & Zhang, 2017); therfore, surfactant Cremophor EL and PEG 400, as well as organic solvent ethanol, were employed to solubilize ICT in water. C_max_ and T_max_ were 541.1 ng/ml and 4 hr for ICT, and 4,236.7 ng/ml and 2 hr for GICT, respectively. C_max_ value of 1617 ng/ml appeared at 2 hr when multiple doses of ICT were intraperitoneally injected to rats daily for 7 consecutive days at a dose level of 40 mg kg^‐1^ day^‐1^ (Zhang, 2014). AUC_0‐24h_ ratio of GICT to ICT was 5.92 and concentration ratio range of GICT to ICT at each corresponding time point was 1.0–18.8, implying that ICT was predominantly and rapidly biotransformed to GICT although ICT absorption was slow, and it was necessary to evaluate the safety of GICT in vivo. GCIT was slowly eliminated in vivo with a terminal half‐life (t_1/2, λz_) value of 4.51 hr, and t_1/2, λz_ value of ICT was 3.14 hr. For intraperitoneal administration, t_1/2, λz_ values of ICT and GICT in plasma were much shorter than those in liver, spleen, kidney, lung, muscle, adipose, and brain in the range of 8.80–50.56 hr (Zhang, 2016). t_1/2, λz_ value of intraperitoneal route fell between those of intravenous route o(1.72 hr) and oral route (7.37 hr) (Zhang & Zhang, 2017). Similarly, systemic clearance (Cls) and volume of distribution (V) of intraperitoneal administration were between those of intravenous administration and oral administration (Zhang, 2016). In others’ studies (Chang et al., 2012; Wong et al., 2009), conjugated ICT, a phase II metabolite of ICT, was misused as a part of total ICT to calculate pharmacokinetic parameters. The LLOQ value (20 ng/ml) was too high to detect the plasma ICT concentration failed after 2 hr postdosing (Chang et al., 2012). In our pharmacokinetic study, a parent compound ICT (not conjugated ICT) was quantified at a very low concentration based on a small amount of plasma using the present method. The genuine pharmacokinetic properties of ICT were achieved for the evaluation of the compound in animals.

## 4 CONCLUSION

A reliable UPLC‐MS/MS method was firstly established for the quantification of ICT and its dominant metabolite GICT in plasma. The linearities were acceptable for ICT (*r* = 0.9960) and GICT (*r* = 0.9968), and LLOQ values were 0.5 and 5 ng/ml, respectively. The accuracy fell in the range of 92.0%–103.1%, and precision was within 9.5%. The UPLC‐MS/MS method performed well in linearity, accuracy, precision, and recovery and was firstly utilized for pharmacokinetic and metabolism studies of ICT in female rats following a single intraperitoneal injection of ICT. ICT was predominantly and rapidly biotransformed to GICT which was slowly eliminated in vivo with a terminal half‐life value of 4.51 hr.

## AUTHOR CONTRIBUTIONS

Shuang‐Qing Zhang designed the experiments; Zhen‐Wu Huang, Yue‐Xin Yang, and Ling‐He Huang performed the experiments; Zhen‐Wu Huang wrote the manuscript. All authors revised and approved the manuscript.

## ETHICAL APPROVAL

The animal experiments were approved by our Institutional Animal Care and Use Committee (approval number: R00180502) and were performed in accordance to the Guide for the Care and Use of Laboratory Animals (NIH publication no. 85–23, eighth edition in 2011).
